# An NC-AFM and KPFM study of the adsorption of a triphenylene derivative on KBr(001)

**DOI:** 10.3762/bjnano.3.25

**Published:** 2012-03-12

**Authors:** Antoine Hinaut, Adeline Pujol, Florian Chaumeton, David Martrou, André Gourdon, Sébastien Gauthier

**Affiliations:** 1 CNRS, CEMES (Centre d'Elaboration des Matériaux et d'Etudes Structurales), BP 94347, 29 rue Jeanne Marvig, F-31055 Toulouse, France

**Keywords:** atomic force microscopy, insulating surfaces, Kelvin force probe microscopy, molecular adsorption

## Abstract

The adsorption on KBr(001) of a specially designed molecule, consisting of a flat aromatic triphenylene core equipped with six flexible propyl chains ending with polar cyano groups, is investigated by using atomic force microscopy in the noncontact mode (NC-AFM) coupled to Kelvin probe force microscopy (KPFM) in ultrahigh vacuum at room temperature. Two types of monolayers are identified, one in which the molecules lie flat on the surface (MLh) and another in which they stand approximately upright (MLv). The Kelvin voltage on these two structures is negatively shifted relative to that of the clean KBr surface, revealing the presence of surface dipoles with a component pointing along the normal to the surface. These findings are interpreted with the help of numerical simulations. It is shown that the surface–molecule interaction is dominated by the electrostatic interaction of the cyano groups with the K^+^ ions of the substrate. The molecule is strongly adsorbed in the MLh structure with an adsorption energy of 1.8 eV. In the MLv layer, the molecules form π-stacked rows aligned along the polar directions of the KBr surface. In these rows, the molecules are less strongly bound to the substrate, but the structure is stabilized by the strong intermolecular interaction due to π-stacking.

## Introduction

The study of molecular adsorption on atomically clean, well-defined surfaces of bulk insulators is progressing rapidly due to the development of atomic force microscopy in the noncontact (or frequency modulation) mode [1–13]. A wide variety of structures, from 3-D islands to single molecules have been observed on different surfaces with an ever-increasing resolution, and there is still room for progress as shown by the impressive submolecular resolution that has been demonstrated in recent works on the adsorption of pentacene [14] or decastarphene [15] molecules on Cu(111) and on a NaCl(001) bilayer on Cu(111). During the same period, Kelvin probe force microscopy (KPFM) has been combined with NC-AFM [16–19] to investigate metallic or semiconducting surfaces, as well as adsorbates [20–21] or thin insulating films on metals [18,22–23]. But its application to bulk insulating surfaces [24–26] is only beginning, and studies of molecular adsorption on these surfaces are still very scarce [6,8]. Coupling these two techniques is not only interesting for the characterization of the electrical properties of the adsorbates, but also for the extraction of topographic images that are free from distortion induced by electrostatic forces [27].

**Figure 1:**
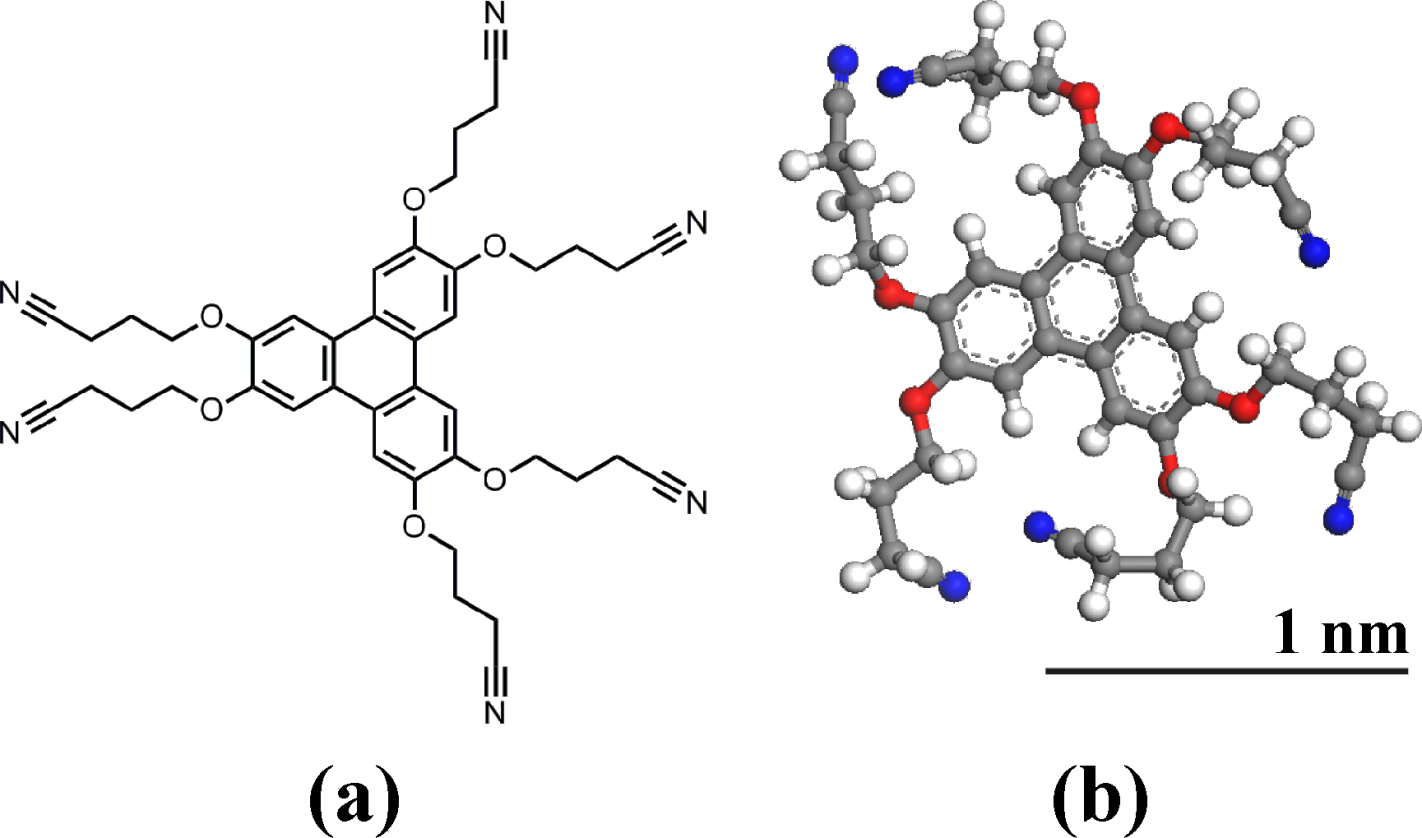
(a) Molecular scheme and (b) structure of HCPTP optimized in vacuum.

In the following, we present the first results of a coupled NC-AFM and KPFM study of the adsorption on KBr(001) of 2,3,6,7,10,11-hexa(cyanopropyloxy)triphenylene (HCPTP), presented in Figure 1. This molecule was designed to adsorb strongly on an alkali halide surface in the hope of blocking its diffusion at room temperature. It is equipped with six flexible propyl chains ending with dipolar CN groups. These groups were proven recently to behave as strong anchoring entities for a truxene derivative adsorbed on KBr(001) [10–11] due to their efficient electrostatic interaction with the K^+^ ions of the surface.

## Results

### Low coverage deposits

**Figure 2:**
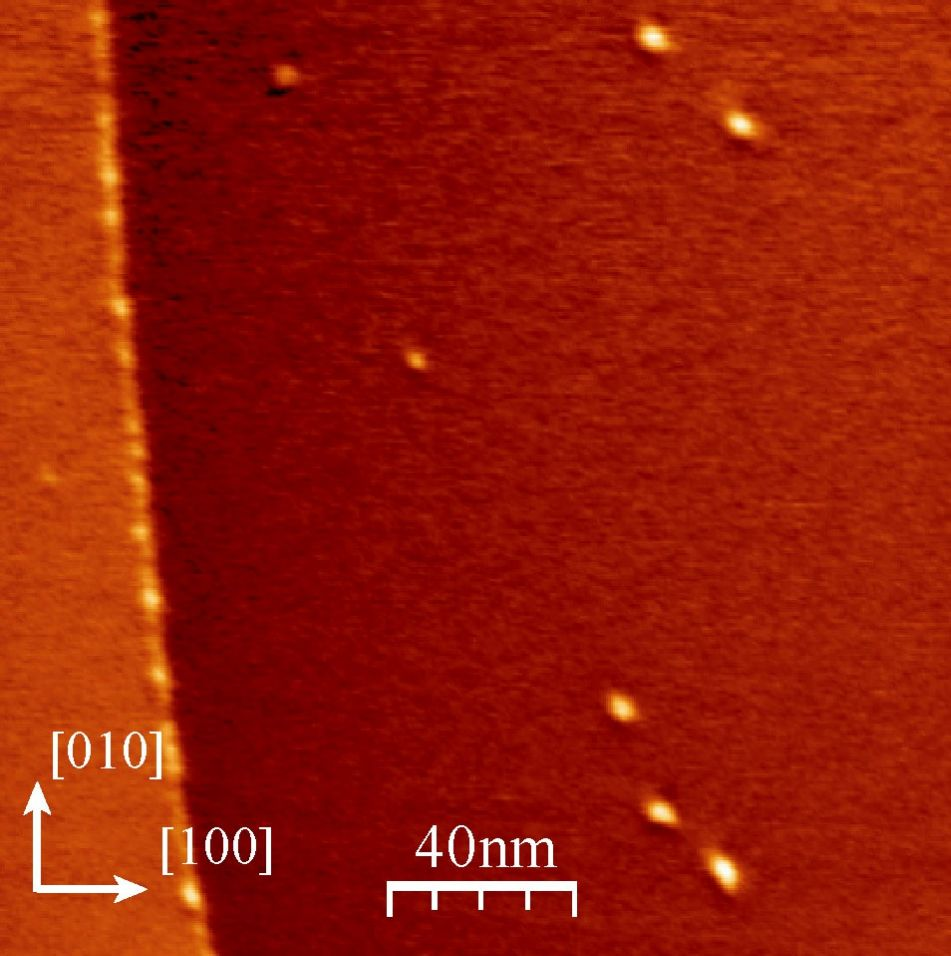
Constant-frequency-shift image of the KBr surface after the deposition of a small amount of molecules at room temperature. Imaging conditions: Δ*f* = −5 Hz, oscillation amplitude *A* = 2 nm.

An image of the molecules at low coverage, deposited at room temperature on KBr(001), is shown in Figure 2. The white dots that appear on the step edge have a size that is compatible with single molecules, but the resolution is not high enough for a convincing identification. The dots on the terraces are more extended. Their diameter ranges from 3 to 5 nm whereas their height reaches 0.9 nm. We interpret them to be small molecular aggregates comprising a few to a few tens of molecules.

**Figure 3:**
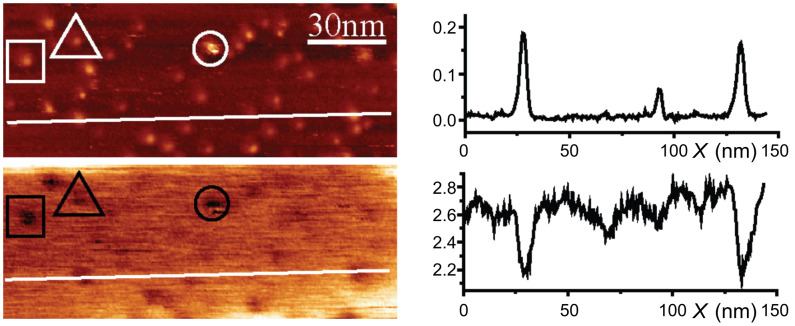
Upper image: topography and lower image: Kelvin map of a KBr terrace with a higher coverage. Imaging conditions: Δ*f* = −20 Hz, *A* = 2 nm. The profiles correspond to the white lines drawn on the images.

A KBr terrace with a higher coverage is shown in Figure 3, with its simultaneously measured Kelvin voltage map. A negative shift of approximately 0.4 V appears on the largest aggregates relative to the mean KBr signal. According to the standard interpretation [28–30], the sign of this shift is indicative of the presence of permanent dipoles that have a component pointing outward from the surface, or of positive charges under the tip.

### Higher coverage deposits

**Figure 4:**
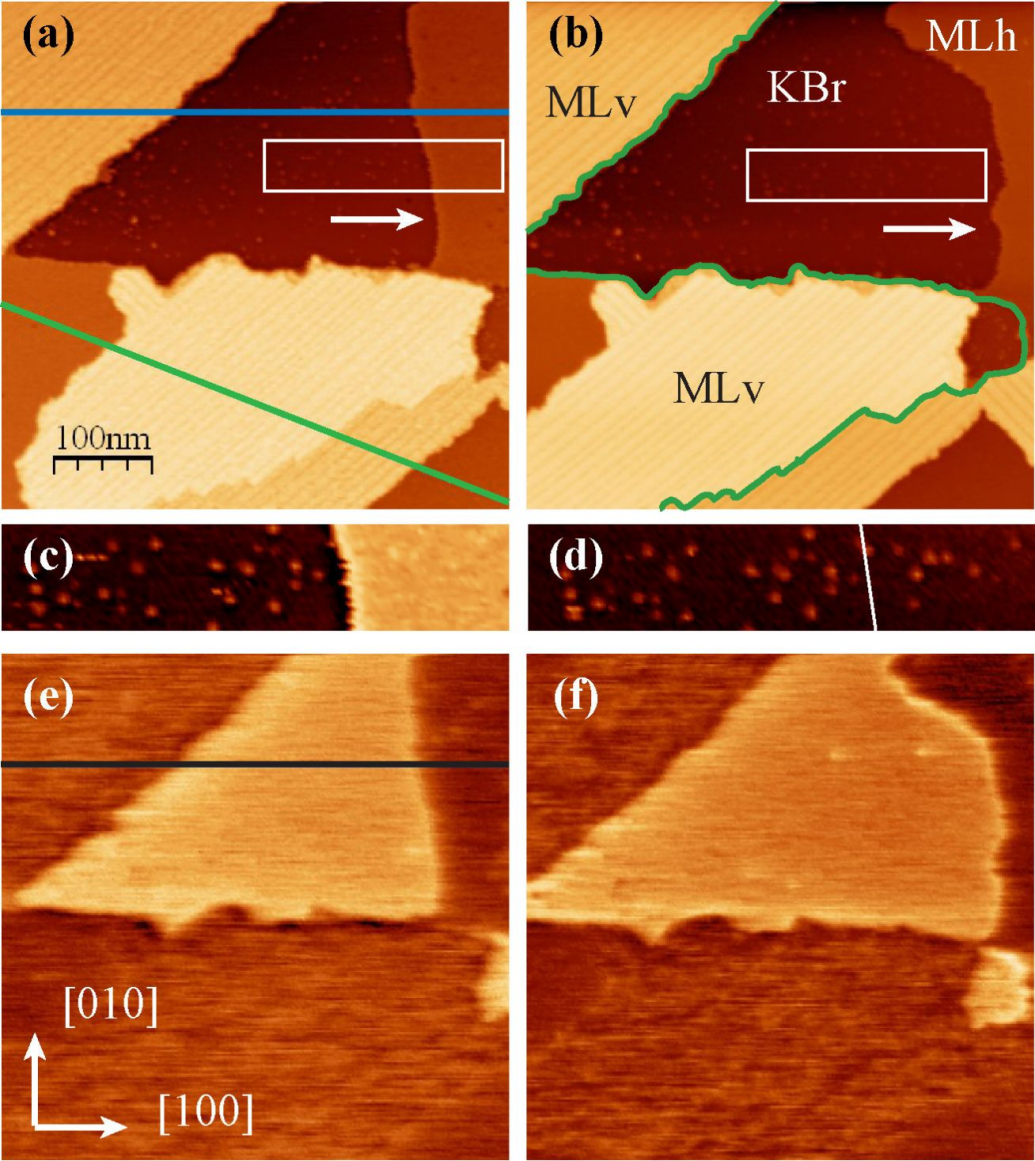
Images of a sample annealed at 150 °C after the deposition of the molecules at room temperature. (a) and (b): topographic images, (c) and (d): enlargement of the areas marked by a white rectangle on (a) and (b); (e) and (f): Kelvin maps obtained simultaneously with (a) and (b). (b), (d) and (f) were measured on the same area as (a), (c) and (e) after a time lapse of 13 h. Imaging conditions: Δ*f* = −20 Hz, *A* = 2 nm. Two KBr monoatomic steps are outlined in green in (b).

For higher coverage, the molecules were deposited at room temperature and the surface was studied at room temperature and after annealing at 80 °C and 150 °C. The complete study of the evolution of this system with temperature is not the purpose of this report. Here, for the sake of simplicity, we only discuss the results after annealing at 150 °C. Note that the types of structure we observed after the 150 °C annealing were already present at room temperature, differing essentially by the size of their domains. The images of a higher-coverage deposit, annealed at 150 °C during 30 min, presented in Figure 4, show that several structures coexist on the surface.

**Figure 5:**
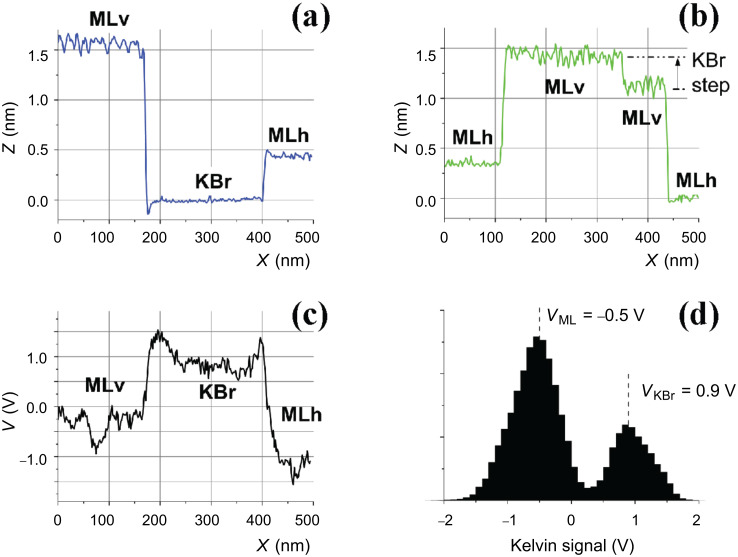
(a) and (b) are the profiles that correspond to the blue and green lines drawn in Figure 4a; (c) is the profile corresponding to the black line on Figure 4e, and (d) is the histogram of Figure 4e.

We first focus on the upper part of the images. It can be seen by comparing Figure 4a and Figure 4b that the line bordering the large triangular area on its right (arrows in Figure 4a and Figure 4b) has been displaced toward the right during the 13 h time lapse separating them. The enlargements of Figure 4c and Figure 4d show that the surface liberated by this process presents dots that are quite similar to the molecular aggregates of Figure 3. For this reason, we identify this region as the KBr substrate. The phenomenon observed in Figure 4 can then be attributed to a dewetting process of a molecular layer [4] corresponding to the domain labeled MLh in Figure 4a. Its height of 0.4 nm (profile in Figure 5a) is compatible with the height of a molecule lying flat on the substrate. Observation of such an evolution at room temperature on a system that has been annealed at 150 °C shows that it is kept far from equilibrium by the very slow kinetics of reorganization. The Kelvin maps of Figure 4e and Figure 4f show a very clear contrast between KBr and MLh (profile in Figure 5c). The Kelvin bias on MLh is shifted toward negative values relative to KBr, that is, in the same direction as for the aggregates discussed previously.

We now examine the third type of domain present in these images (labeled MLv), which displays dark lines oriented along a polar <110> direction (see also Figure 8). The profiles of Figure 5a and Figure 5b show that their height relative to the KBr surface amounts to approximately 1.6 nm. This value does not vary from one layer to the other (note that the domain located in the lower part of Figure 4a and Figure 4b is crossed by a KBr monoatomic step, outlined in Figure 4b) and is comparable to the diameter of a molecule (see the scale in Figure 1). This observation indicates that these MLv domains comprise a layer of molecules standing approximately upright on the KBr surface. The mean distance between the dark lines is around 11 nm and the associated corrugation is between 0.1 and 0.2 nm. The Kelvin maps of Figure 4e and Figure 4f show that the Kelvin signal is also shifted toward negative values relative to KBr for the MLv domains, with a comparable mean Kelvin bias (histogram in Figure 5d). Nevertheless, the Kelvin map on the two types of structures has a different aspect, being more heterogeneous on the MLv domains. This heterogeneity is not clearly correlated to the topographic images.

**Figure 6:**
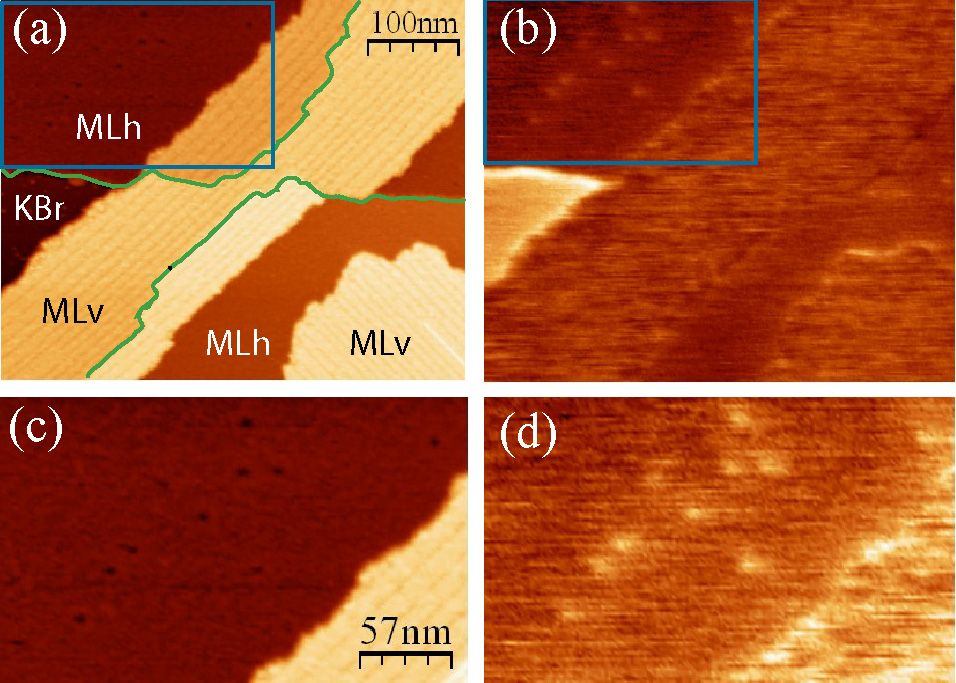
(a) Topographic and (b) Kelvin map of a high molecular coverage annealed to 150 °C; (c) and (d) are enlargements of the upper-left parts of (a) and (b) (blue rectangle). Δ*f* = −20 Hz, *A* = 2 nm.

Another example of images of a high-coverage deposit, annealed to 150 °C, is shown in Figure 6. Some well-resolved defects appear in the MLh domain in the upper-left area of the topographic image of Figure 6a (see the enlargement in Figure 6c). These defects are also visible in the Kelvin map of Figure 6b (enlarged in Figure 6d). They appear as clear dots, corresponding to a positive shift of the Kelvin voltage on the order of 0.8 V relative to the mean Kelvin voltage of the surrounding MLh domain. As expected the spatial resolution in the Kelvin map is lower than in the topography map due to the longer range of electrical forces relative to van der Waals forces.

The different domains that appear in Figure 6 have been labeled and the monoatomic KBr steps outlined in green. These attributions are based on the measurement of the height of the different structures and their Kelvin signature, as discussed previously. The steps have a remarkable shape, quite different from what is observed on the clean KBr surface, before adsorption, where they are mostly straight and aligned along the nonpolar KBr(001) directions. It is clearly seen that the step morphology is strongly coupled to the structure of the MLv domains. The steps tend to align along the same polar direction as the dark lines of the structure. These steps are highly unstable on the clean surface due to their high electrostatic energy. They can be stabilized only by charged species, in the present case by adsorption of the negatively charged N atoms of the CN groups. This observation also points to a massive KBr surface mass transfer during the annealing of the substrate due to molecular adsorption. The mechanisms at work during this transformation could be of the same nature as those discussed recently in the study of the restructuring of KBr(001) steps by truxene molecules [11]. Finally, we note that when the molecular structure crosses portions of steps that are not aligned in these directions, these dark lines are not visibly affected, indicating that this structure has a strong intrinsic cohesion.

### High-resolution images of MLh and MLv domains

**Figure 7:**
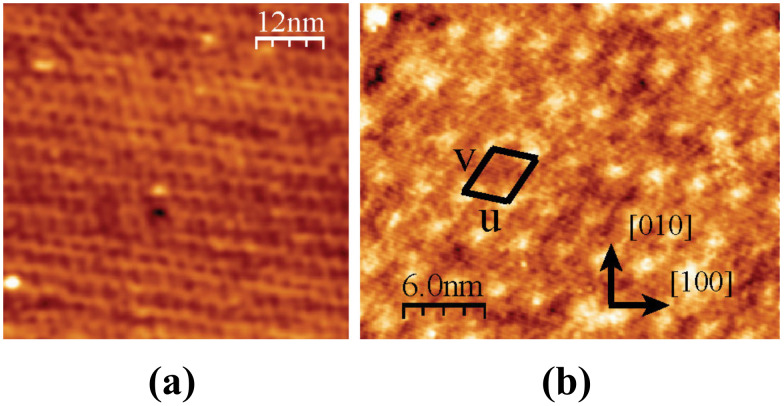
High resolution topographic images of an MLh domain. *A* = 2 nm. (a) Δ*f* = −35 Hz, (b) Δ*f* = −50 Hz. The unit cell is indicated in (b).

Two high-resolution images obtained on the same MLh domain are shown in Figure 7. The molecular network can be described by a unit cell characterized by *u* (2.9 nm, −13° from [100]) and *v* (3.7 nm, +61° from [100]) (Figure 7b). Note that due to the different imaging conditions in Figure 7a and Figure 7b, the molecular layer appears as a network of black holes in (a) and white bumps in (b). Comparing the size of this unit cell with the size of the molecule (Figure 1) suggests that the basis of the network comprises two molecules.

**Figure 8:**
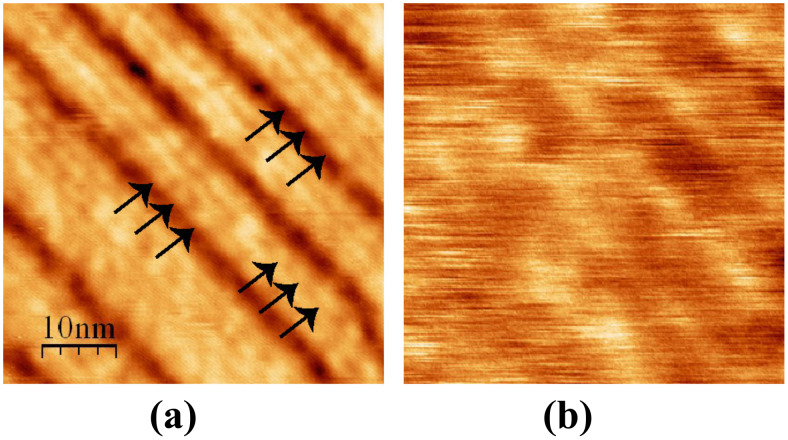
High resolution (a) topographic and (b) Kelvin image of an MLv domain. *A* = 2 nm, Δ*f* = −20 Hz. The arrows in (a) point to the ~4 nm modulation.

The images on a MLv domain displayed in Figure 8 show that the dark lines that appear in the large-scale images of Figure 4 and Figure 6 are separated by thinner lines, delimiting rows with a width of ~2.3 nm, slightly larger than a single molecule. A modulation with a period of ~4 nm appears along the rows. Because of the above-mentioned observation that the dark lines can cross a KBr atomic step without be perturbed, we tentatively interpret these observations as indicating that one row corresponds to a stack of molecules in relatively strong interaction. Note that, as remarked before, the Kelvin map (Figure 8b) is very heterogeneous, with values of the Kelvin voltage varying between −0.5 and +1.2 V. This dispersion is indicative of a certain degree of disorder as is also observable in the topography image of Figure 8a.

### Numerical simulations

**Figure 9:**
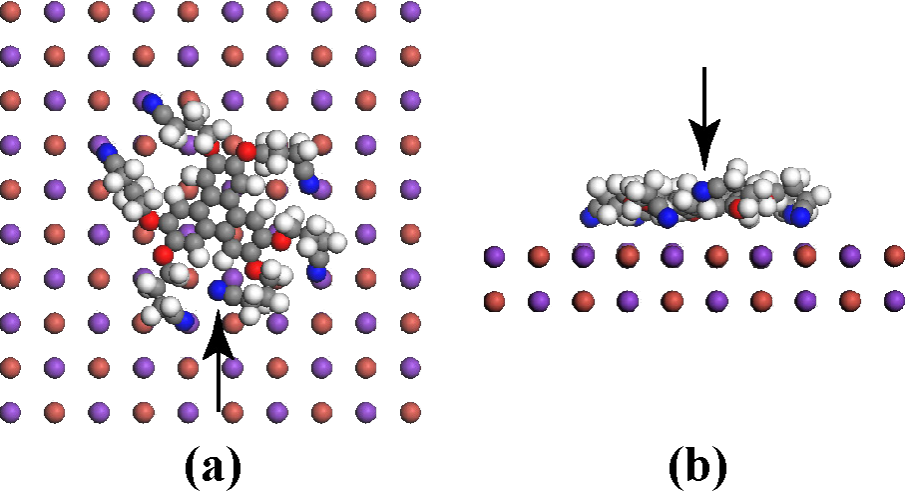
Lowest-energy adsorbed conformation of HCPTP adsorbed on KBr(001). (a) Top and (b) side view. K^+^ ions are violet, N atoms are blue. The arrow in (b) points to the CN group that is not bound to a K^+^ ion.

To gain insight into the adsorption and dynamic properties of HCPTP on KBr(001), we performed numerical simulations, as described in the Methods section. The calculated lowest-energy adsorbed conformation of HCPTP on KBr(001) is displayed in Figure 9.

The molecule is bound to the surface by the electrostatic interaction between its CN groups and K^+^ ions. The flexibility of the propyl chains allows the molecule to reach five K^+^ ions. One of the chains cannot bind and its CN group stays at a larger distance from the surface (Figure 9b). The N atoms of the CN groups that bind the molecule are at a mean distance of 0.28 nm while the central aromatic core lies flat at a distance of 0.4 nm from the surface plane. The calculated adsorption energy of 1.8 eV is quite large. It includes not only the contribution of the five CN groups but also the interaction energy of the negatively charged oxygen atoms and the aromatic core with the surface, which can be roughly evaluated by calculating the adsorption energy of hexamethoxytriphenylene on KBr(001) under the same conditions. We obtain 0.8 eV, meaning that each CN group contributes approximately (1.8 − 0.8)/5 = 0.2 eV, in good agreement with the value obtained for the CN groups of the truxene derivative mentioned previously [10].

Molecular dynamics studies of the diffusion of a HCPTP molecule were performed with the same force field in the NVT ensemble with a Nose–Hoover thermostat. Simulations at 300 K show that the molecules diffuse by successive hopping of CN groups from one K^+^ to another in a way that is similar to the "walking" of the truxene-derived molecule described recently [10]. To get an order of magnitude for the diffusion coefficient, we observe that the molecule travels approximately 1 nm in a time *T* = 2.5 ns. Thus, *D* = <*x*^2^>/(4*T*) ≈ 10^−10^ m^2^·s^−1^.

## Discussion

### Molecular structures

Figure 2 shows that step edges act as preferred adsorption sites for the molecules at low coverage. This is not surprising as the interaction of a CN group is expected to be enhanced due to the availability of adjacent K^+^ sites on nonpolar steps, as shown for truxene molecules [11]. The origin of the molecular aggregates observed in Figure 2 and Figure 3 is less clear. As indicated previously a single molecule diffuses at room temperature despite its six CN anchoring groups. A lower estimate of the distance that a molecule would travel on a defect-free surface without interacting with another molecule during the deposition can be obtained in the following way: For a deposition molecular flux *F*, the mean time τ_F_ separating the arrival of two successive molecules in an area *L*^2^ is τ_F_ = 1/(*F*·*L*^2^). During this time, a molecule travels a mean distance of <*L*^2^> = 4*D*τ_F_. Combining these relations, we get (<*L*^2^>)^1/2^ ≈ (4*D*/*F*)^1/4^ = 1 μm (with *F* = 3·10^14^ molecules·m^−2^·s^−1^, corresponding to one MLh monolayer in 1000 s). This value is a lower estimate since landing in the same area is a necessary, but far from sufficient, condition for two molecules to meet. Considering that the mean distance between defects on KBr(001) is smaller than 1 μm, this estimation shows clearly that the nucleation is heterogeneous, i.e., dominated by the adsorption of the molecules on defects. Another observation, which points in the same direction, is that the aggregates have a height that is larger than the 0.4 nm of the MLh layer, i.e., they are 3-D. This transition to 3-D, which happens neither for the MLh nor for the MLv, should be favored by a particular adsorption configuration of the molecules adsorbed on the defects responsible for the nucleation of the aggregates.

Note that the density of aggregates observed in Figure 2 and 3 is much higher than the density of defects that are observed on the clean KBr surface before adsorption. Associating each aggregate with one or several defects implies that the molecules are able either to create defects or to combine with preexisting defects, which are mobile at room temperature and consequently undetectable on the images of the clean surface. Investigating precisely the first stages of the growth of this system would answer these questions, but our present data set does not allow conclusions to be drawn. More extensive numerical simulations would also be necessary.

The measurement of a 0.4 nm height for the MLh monolayer and the high-resolution images of Figure 7 are indicative of a structure composed of molecules lying flat on the surface. The observation of the dewetting of this monolayer (Figure 4) shows that the molecules are mobile at the border of the layer. These observations suggest an adsorption geometry close to the calculated conformation in Figure 9. Indeed, considering the high value of the adsorption energy, one does not expect the lateral intermolecular interactions to be strong enough to significantly affect the adsorption conformation of the single molecule.

**Figure 10:**
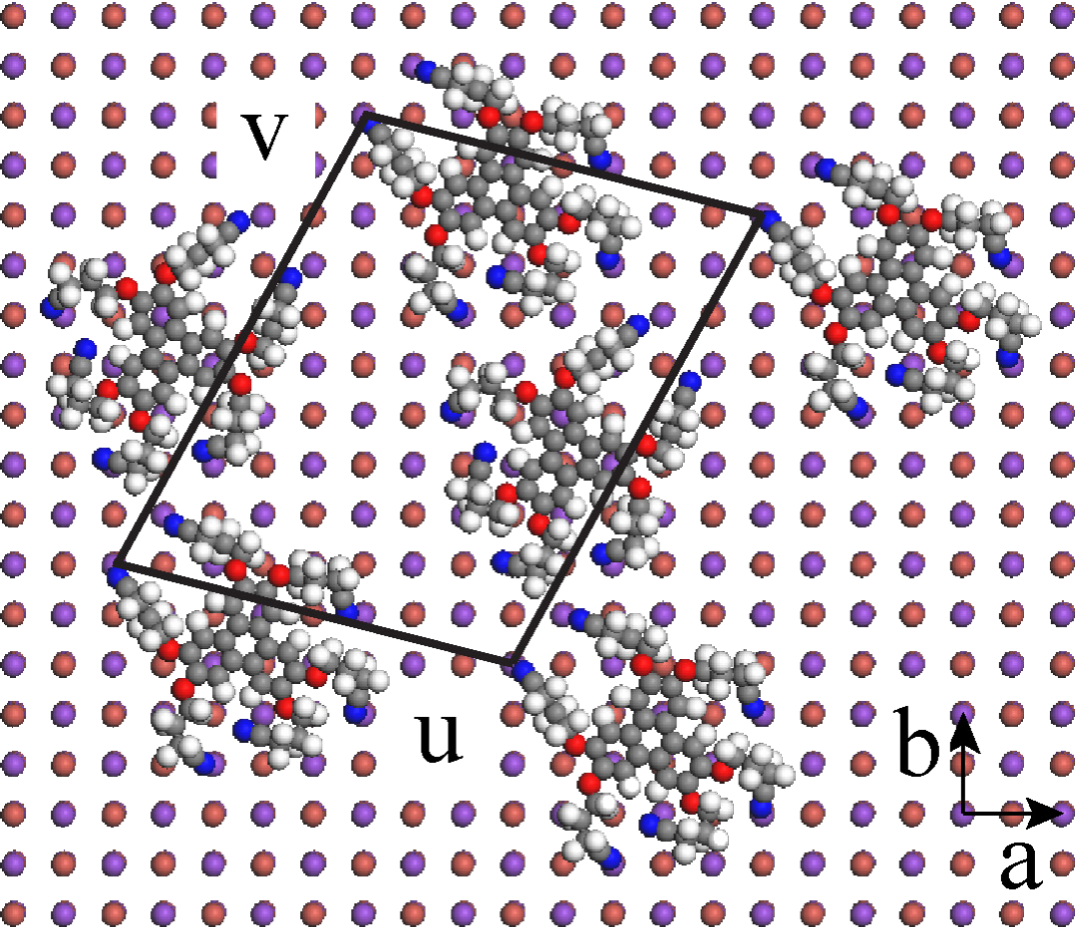
Tentative model of the MLh layer.

A tentative model of the MLh layer is shown in Figure 10. The unit cell vectors *u* and *v* have been chosen on the basis of the experimental values extracted from Figure 7b. They are given in terms of the conventional surface unit-cell vectors *a* and *b* by *u* = 4*a* − b and *v* = 2.5*a* + 4.5*b*. Their modulus and angle are then given by *u* (2.7 nm, −14°/[100]) and *v* (3.4 nm, +61°/[100]) in good agreement with the experiment (Figure 7b). The molecular basis comprises two molecules that have been positioned on the surface in the lowest-energy conformation of Figure 9. Note that the molecules are separated enough to avoid van der Waals contact and should be only weakly interacting with each other, confirming our previous suggestion.

The images of Figure 4 do not show where the molecules of the dewetting MLh layer go. Nevertheless, the fact that the MLh layer was never observed to expand and that no other structures (such as double MLh layers) appeared during this phenomenon strongly suggests that they contribute to the growth of the MLv layer. This conclusion would imply that the MLv structure is more stable than the MLh structure. Considering the structure of the molecule, it is seen that only two CN groups can adsorb on K^+^ ions when the molecule is nearly vertical, contributing approximately 0.4 eV to the adsorption energy. The stabilization of MLv relative to MLh should then result from the intermolecular interaction energy, which should exceed 1.8 − 0.4 = 1.4 eV. This is indeed the case. The interaction energy between two free HCPTP, calculated with the COMPASS force field when the triphenylene cores are in a π-stacking configuration, is on the order of 2.5 eV, well above this threshold. MLv is then more stable than MLh and the transition between these two structures is kinetically limited, as the dewetting transition observed in Figure 4 confirms.

### Interpretation of the observed Kelvin voltages

In the adsorption configuration shown in Figure 9, the molecule acquires a dipolar moment of 12 debyes pointing along the normal to the KBr surface. It is the interaction of the cyano groups with the K^+^ ions that polarizes the molecule. According to the standard interpretation [28–30], such a dipole is expected to induce a negative shift of the Kelvin voltage. Consequently and considering that, as discussed previously, the adsorption conformation of the molecule in the MLh layer should not be significantly different from the single-molecule conformation, we interpret the negative shift of the Kelvin voltage observed on MLh relative to KBr as being due to the adsorption-induced polarization of HCPTP.

The Kelvin contrast on the aggregates is also negatively shifted relative to KBr, indicating that they exhibit either a dipole moment pointing along the KBr surface normal or a positive charge. The contrast on MLv is difficult to interpret without the help of a suitable model. A tentative explanation would be the following: The vertical molecule is adsorbed on two K^+^ ions through two CN groups. This geometry leads to a high density of dipoles pointing along the KBr surface normal when the molecules are stacked in rows. The upper part of the adsorbed molecules, far from this polarized interface, is disorganized, as revealed in particular in the Kelvin maps of this structure, and it is reasonable to assume that due to this randomness it contributes less to the Kelvin voltage. The tip feels dominantly the dipolar layer created by the adsorption of the CN groups on the K^+^ ions. Note that a rapid evaluation gives a dipole density for the MLv that is twice that of MLh, but the precise orientation as well as the distance from the tip should be taken into account for a meaningful comparison of the Kelvin voltage on these two structures.

These considerations were aimed at explaining *the sign* of the shifts of the Kelvin voltage between the different structures. We now comment on *the magnitudes* of the Kelvin voltages that we observe. It has been suggested recently that the mean Kelvin voltage monitored on Kelvin maps can be related to the electrostatic nature of the tip (neutral, polar or charged) [31]. We observed occasionally abrupt variations of the Kelvin voltage (~100 mV), which signal evolutions of the tip structure, but these events were never accompanied by a significant change in the Kelvin contrast as reported in [31]. We conclude that our tips always had the same electrostatic behavior. We also measured slow drifts in the mean Kelvin voltage on a scale of a few volts around 0 V over a time scale of hours. For the three tips we used in these experiments (two Si uncoated tips and a Pt-coated tip) the Kelvin voltage difference between KBr and MLh/MLv was approximately constant at 1.4 ± 0.2 V. These relatively high values are in contrast to the values reported for studies of adsorbates on metals [20–21]. On these systems, the electric potential is fixed at the surface of the metal, at a microscopic distance from the tip. The situation is radically different on thick insulators, in which case the potential is imposed on the metallic plate that supports the sample, which is some millimeters away. Then, the potential drops in the insulator in a way that depends not only on the dielectric constant of the material and the tip–surface distance, but also on the tip radius. The effective potential applied to the surface structures is then largely smaller than the applied bias. This effect, which explains the high values we observe, renders a quantitative analysis of the Kelvin voltage more difficult on bulk insulators than on conducting substrates.

## Conclusion

We have demonstrated that HCPTP forms two types of monolayer on KBr(001): MLh where the molecules are lying flat on the substrate and MLv where they stand upright. High-contrast, well-resolved Kelvin maps were obtained simultaneously with the topographic NC-AFM images. The precise structure of these two monolayers could not be determined from the images. But the measurement of their height on the electrostatic force-compensated topographic images, completed by numerical calculations of the adsorption conformation of the molecule leads to a consistent interpretation of the Kelvin maps in terms of adsorption-induced polarization of the molecule by the electrostatic interaction of the cyano groups with K^+^ ions.

HCPTP was designed with its six CN groups to limit the diffusion of the molecule on the KBr(001) surface. What the present study demonstrates is that maximizing the adsorption energy does not necessarily imply a low diffusion coefficient. There is in fact no simple relation between adsorption and diffusion energy, especially for large molecules with numerous degrees of freedom [32]. Moreover, a high adsorption energy is likely to induce surface restructuring, as observed in the present case. While such processes could be useful to modify the surface at will, this is not always desirable. Clearly, more elaborate strategies are needed to progress toward our objective of immobilizing a molecule on a bulk insulating substrate at room temperature.

## Methods

### 

#### Experimental

Experiments were conducted in a commercial room-temperature (RT) ultrahigh vacuum STM/AFM (Omicron NanoTechnology GmbH, Taunusstein, Germany). The original optical beam-deflection system was improved by replacing the LED by a superluminescent laser diode (Superlum, Moscow, Russia) coupled to the system by an optical fiber. The control electronics were from Nanonis (SPECS, Zurich, Switzerland). The KBr crystal (~3 mm thick) was cleaved in air, quickly transferred to the UHV system and finally heated at 480 K for 1 h to remove the charges produced during the cleavage process. This preparation method produces an atomically well-ordered surface with (001) terraces separated by atomic steps, mainly oriented along the nonpolar <100> crystallographic directions of the surface. We characterized this surface by KPFM and found that charges are always present on the step edges, as reported in [25]. In contrast, only a few charged defects (less than 10 per μm^2^) were observed on the terraces. Molecules were deposited from a heated boron nitride disk on which a few drops of the molecular solution were left to dry. QNCHR silicon cantilevers provided by NanoSensors (Neuchatel, Switzerland) were used, with no special preparation except a moderate heating (150 °C) in vacuum. The resonance frequencies were close to 290 kHz, with quality factors ranging from 40,000 to 45,000. An experiment was also performed with a Pt-coated tip (PtNCH), but the resolution obtained (in topography as well as in the Kelvin maps) was not satisfying. KPFM coupled to NC-AFM exists in two flavors [17], namely the frequency- and amplitude-modulation modes. In this work, we use the frequency-modulation mode. The voltage was modulated at 1 kHz with an amplitude of 2 V. The images were obtained in the constant Δ*f* mode with an oscillation amplitude of *A* = 2 nm and small values of Δ*f*, corresponding to normalized frequency shifts [33] γ = *kA*^3/2^Δ*f*/*f*_0_ = 0.25 fN·m^1/2^ at most (*k* ~ 40 N·m^−1^). Under these conditions the interaction of the tip with the surface is quite small and it is expected that only van der Waals and electrostatic forces contribute to the image. Note that at the Kelvin voltage, provided that no net surface charges are present, the topographic image becomes a pure van der Waals image. The measured height of the observed structures is then close to that given by the molecular models, facilitating the structural identification. Great care was taken to avoid any cross-talk between the different signal channels available in NC-AFM. Constant Δ*f* images were recorded simultaneously with maps of the Kelvin voltage, the frequency shift, the amplitude and the excitation voltage. For all the images presented in this paper, the excitation voltage map was uniform, at a value close to its value in the absence of tip–surface interaction. The <100> nonpolar directions of the KBr(001) surface are oriented in the horizontal and vertical directions.

#### Molecular synthesis

2,3,6,7,10,11-Hexa(cyanopropyloxy)triphenylene (HCPTP) was prepared in 73% yield by reaction of 2,3,6,7,10,11-hexahydroxytriphenylene (HHTP) with 4-bromobutyronitrile in dry DMF in the presence of potassium carbonate. HHTP was obtained from 2,3,6,7,10,11-hexamethoxytriphenylene according to [34]. To a suspension of 4-bromobutyronitrile (636 mg, ca. 0.43 mL, 4.3 mmol) and dry potassium carbonate (3.2 g, 23.2 mmol) in dry DMF under argon was added HHTP (200 mg, 0.62 mmol). The mixture was then stirred under argon at RT for 55 h and then poured into water (150 mL) under stirring. Neutralization by H_2_SO_4_ (5 M) gave a beige precipitate, which was filtered and dried under vacuum. Recrystallization from ethyl acetate (300 mL) or by column chromatography (SiO_2_, DCM–AcOEt, 8:2) and drying under vacuum at 40 °C for 7h gave HCPTP (319 mg, yield: 71%). *R*_f_ = 0.5 (TLC, DCM–AcOEt, 7:3); ^1^H NMR (CD_2_Cl_2_, 300 MHz) δ 7.90 (s, 6H, arom), 4.36 (t, *J =* 5.7 Hz, 12H, *CH*_2_O), 2.71 (t, *J* = 7 Hz, 12H, *CH*_2_CN), 2.27 (m, 12H, NCCH_2_*CH*_2_) ppm; ^13^C NMR (CD_2_Cl_2_, 75 MHz) δ 149.1, 124.4, 108.1, 67.8, 26.2, 14.9 ppm; MS *m*/*z* (DCI, NH_3_): 728 [M + H]^+^; Anal. calcd for C_42_H_42_N_6_O_6_: C, 69.4; H, 5.8; found: C, 69.2; H, 6.1.

#### Numerical simulation

The geometry of the molecule adsorbed on KBr(001) was optimized by using Materials Studio [35] with the COMPASS force field [36]. This force field is well adapted to the system that we consider here, because it has been parameterized by using condensed-phase properties in addition to ab initio and empirical data for isolated molecules [37–38]. It is well known from previous studies that the adsorption of organic molecules on this type of surface is dominated by van der Waals and electrostatic interactions, and that the charge transfer between the substrate and the molecule is negligible [39]. The KBr slab was composed of 6 × 6 × 3 unit cells. Two KBr layers were free to relax during the simulations.
